# Saroglitazar suppresses KIM-1 and type IV collagen in high fat diet and low-dose streptozotocin-induced diabetic nephropathy in Wistar rats

**DOI:** 10.22038/ijbms.2024.78221.16908

**Published:** 2024

**Authors:** Rizwan Ahamad, Uma Bhandari, Sayima Nabi, Shweta Sharma

**Affiliations:** 1 Department of Pharmacology, School of Pharmaceutical Education & Research (SPER), Jamia Hamdard, New Delhi-110062, India

**Keywords:** Diabetic nephropathy, Fenofibrate, Kidney injury molecule-1, Saroglitazar, Streptozotocin, Type IV collagen

## Abstract

**Objective(s):**

Nephropathy is the most common comorbidity linked to T2D. The present study aimed to examine the potential of saroglitazar in the context of a high-fat diet and low-dose streptozotocin-induced diabetic nephropathy in Wistar rats.

**Materials and Methods:**

Molecular docking simulation investigations were conducted on the ligand-binding region of type IV collagen and Kidney injury molecule-1 (KIM-1), using saroglitazar and fenofibrate as the subjects. The rats were fed either a conventional rodent diet or a high-fat diet *ad libitum* for two weeks. Following a two-week period, the rats given an HFD were administered with a low-dose of STZ (35 mg/kg, IP). Rats with experimentally induced diabetes were categorized into five groups: normal control; diabetic control; HFD+STZ+saroglitazar (2 mg/kg); HFD+STZ+saroglitazar (4 mg/kg); HFD+STZ+fenofibrate (100 mg/kg) treated orally for 21 days with continuation on HFD. After 21 days, rats were kept on fasting overnight, blood and urine was acquired for various biochemical analysis. Animals were sacrificed, and kidney tissues were removed for histopathological studies.

**Results:**

*In-silico* investigation showed a substantial affinity between saroglitazar and fenofibrate with KIM-1 and type IV collagen. Saroglitazar produced a significant (*P*<0.01) reduction in weight of the body, serum blood sugar, albumin, creatinine, and BUN levels. Further, saroglitazar significantly (*P*<0.01) reduced the KIM-1 and type IV collagen levels in the urine of diabetic rats. Histopathological results showed improvement in tubular degeneration, necrosis, and dilatation of Bowman’s space in kidney tissue.

**Conclusion:**

Saroglitazar attenuated renal injury by improving renal function in HFD+STZ-induced DN in Wistar rats.

## Introduction

Diabetic nephropathy (DN) is a common and serious consequence of diabetes mellitus (DM) that is linked to higher rates of illness and death in individuals with diabetes (1). The prevalence of diabetes was projected to be over 400 million in 2017, and it is estimated to increase significantly to 629 million by 2045 globally. This would substantially burden public health professionals (2-4). DN is distinguished through a gradual escalation in proteinuria, albuminuria, reduction in glomerular filtration rate, hypertension, and a heightened susceptibility to cardiovascular morbidity and death (5). The initial laboratory abnormality is a positive microalbuminuria test. The diagnosis is typically detected when a regular urinalysis of an individual with DN reveals excessive protein in the urine. The urine analysis may also indicate the presence of sugar in the urine, particularly if blood sugar is poorly controlled. As kidney disease worsens, serum blood urea nitrogen (BUN) and creatinine levels may rise. Thus, managing metabolic and hemodynamic variables is essential to stop DN from worsening (6).

Presently, the therapeutic choices for treating patients with DN encompass agents such as ACE inhibitors, SGLT2 inhibitors, AT1 receptor blockers, and some anti-oxidants, which have demonstrated some improvement in kidney function in individuals with diabetes (5, 7). Consequently, there are ongoing efforts to explore innovative and hopeful therapy approaches for treating DN. 

Recently, there has been global interest in dual peroxisome proliferator-activated receptor (PPAR) α/γ agonists. These agonists are nuclear receptors triggered by fatty acids and control metabolic processes by regulating sugar and lipid metabolism in distinct ways. There are three main types of PPARs: PPARα, PPARβ/δ, and PPAR-γ (8). Recent research indicates that peroxisome proliferator-activated receptor alpha (PPARα) agonists have significant promise in treating DN. This is because they can help prevent renal lipid accumulation-induced lipotoxicity, a known risk factor for nephropathy in individuals with chronic DM (9). 

Saroglitazar, a medication that activates both PPAR-α and PPAR-γ receptors, is a highly successful treatment for controlling diabetic dyslipidemia. The intervention yields two advantages: a notable enhancement in glycemic parameters (glycated hemoglobin and fasting blood glucose) and a substantial improvement in dyslipidemia (triglycerides, apolipoprotein B, non-HDL cholesterol) (10). 

Saroglitazar activates PPAR-γ, a protein that has a crucial function in regulating insulin sensitivity and inflammation. Saroglitazar improves insulin sensitivity by activating PPAR-γ, which helps to manage blood sugar levels better. Improved insulin sensitivity can also have additional benefits for renal health by reducing the metabolic burden on the kidneys (11, 12).

KIM-1, often referred to as Kidney Injury Molecule-1, and collagen type 4 are biomarkers associated with kidney injury and fibrosis, respectively (13). KIM-1 is a biomarker that signifies kidney damage and typically rises in reaction to injury to the renal tubules. Studies have shown that saroglitazar can activate PPAR-γ, which may help protect the kidneys from injury and reduce the expression of KIM-1. Saroglitazar can reduce KIM-1 levels by alleviating renal impairment (14).

Type IV collagen has a crucial function in the composition of the extracellular matrix in the renal system. Renal fibrosis, a common clinical feature of DN and alternative forms of chronic kidney disorders, is related to an increased buildup of Type IV collagen. Saroglitazar’s activation of PPAR-gamma may lead to a reduction in the production and buildup of Type IV collagen in the kidneys. There is a possibility that this could decrease renal fibrosis and maintain kidney function (15).

Fenofibrate, an FDA-approved medication, acts as an agonist for PPARα and is used to manage and treat conditions such as hypertriglyceridemia, primary hypercholesterolemia, or mixed dyslipidemia. It works by increasing lipoprotein lipase activity, promoting the synthesis of high-density lipoprotein (HDL), and reducing the liver’s production of apolipoprotein C (16-18). 

However, the impact of saroglitazar on DN has not been explored yet. Therefore, the current research aimed to evaluate the impact of saroglitazar on DN in Wistar rats induced by an HFD and low-dose STZ. Two different doses of saroglitazar (2 mg/kg, PO and 4 mg/kg, PO) were administered, while fenofibrate (100 mg/kg, PO) was used as a reference drug for comparative purposes.

## Materials and Methods

***Drugs and chemicals***

STZ was acquired from Sigma Aldrich (St. Louis, MO, USA). Saroglitazar and fenofibrate were purchased from a local chemist shop in New Delhi, India. All the other compounds used were commercially available and of AR quality.

***Computational studies (Molecular docking)***

Computational studies were carried out to analyze the binding affinities of ligands in the domain of KIM-1 and Type IV collagen using AutoDock Vina 1.5.7 (19). The 3D structure determined by X-ray crystallography of KIM-1 (PDB ID: 5DZO; resolution: 1.30 Å) and Type IV collagen (PDB ID: 1LI1; resolution: 1.90 Å) was obtained from the RCSB Protein Data Bank. The ligands’ structure was shown in Chem Sketch and then transformed into pdb format using the Open Babel program Version (3.1.1) (20).

The proteins were acquired separately and added to Biovia Discovery Studio Visualizer-BDSV for protein preparation. The water molecules attached to the protein and the ligand in the co-crystal were removed, and H-atoms with polarity were introduced. Ligands and proteins were stored in.pdbqt format using AutoDock Vina (MGL Tools 1.5.7). A grid box with dimensions of 40 Å, 40 Å, and 40 Å for the x, y, and z axes, correspondingly, was created to determine the binding region of KIM-1 (PDB ID: 5DZO). The grid center was set at x=56.047 Å, y=33.583 Å, and z=66.446 Å, with a grid spacing of 0.385 Å. A grid box measuring 126 Å in the x, y, and z axes was created. The center point of the grid is located at x=16.768 Å, y=61.425 Å, and z=0.664 Å. The spacing between grids is set at 0.771 Å. This grid was developed to determine the binding region of Type IV collagen (PDB ID: 1LI1). The level of comprehensiveness in both proteins was adjusted to 33. The conformation with the lowest energy was selected as the optimal choice, and the dock position was stored. The ligand interaction design and the docking position were captured utilizing the Discovery Studio Visualizer. 

***Experimental animals***

The study received approval from the Institutional Animal Ethics Committee at Jamia Hamdard, located in New Delhi, India (Approved no. 1860; Dated: 30-12-2022), and was carried out in compliance with the standards set by the Committee for Control and Supervision of Experiments on Animals (CPCSEA), India. The male Wistar albino rats (6–8 weeks/150–200 g) were obtained from the Central Animal House Facility of Jamia Hamdard in New Delhi, India. Rats were kept in hygienic rat cages with solid bottoms of stainless steel mesh polypropylene. They were maintained in a controlled environment with an RT of 22 ± 2 °C and humidity of 55 ± 5%. The rats were subjected to a 12-hr light and dark cycle. The rats had access to conventional rat meal and water *ad libitum*. Rats were provided one week to adjust to the Exploratory surroundings prior to the feeding intervention.

***Induction of T2D with HFD and low-dose STZ***

The rats were divided into two dietary regimens by feeding either conventional rat feed or HFD (from National Institute of Nutrition (NIN), Hyderabad, India) *ad* *libitum* for the starting phase of 2 weeks (21). The composition of HFD was detailed elsewhere (17). Following two weeks of dietary manipulation, the rats fed an HFD received an intraperitoneal injection of a low dose of STZ (35 mg/kg). In contrast, the control rats were administered a vehicle citrate buffer (pH 4.4) in a dosing volume of 1 ml/kg, also via intraperitoneal injection. Fasting blood glucose levels were evaluated three days after administering either the vehicle or STZ injection. Rats with an FBG level of ≥200 mg/dl were classified as diabetic and chosen for pharmacological investigations. The rats were permitted to consume their diets until the end of the study. The animal model has previously been developed in our laboratory (22). 

***Experimental protocol***

A total of 30 male rats, consisting of 6 normal control rats and 24 rats with HFD+STZ-induced diabetes, were involved in the study. The rats were randomly separated into five groups, each including six rats as follows:

Group I: normal control rats were given 0.5% CMC in normal saline daily (NC)

Group II: toxic diabetic rats were given 0.5% CMC daily (TC)

Group III: diabetic rats were given saroglitazar (2 mg/kg/day, PO) 

Group IV: diabetic rats were given saroglitazar (4 mg/kg/day, PO)

Group V: diabetic rats were given fenofibrate (100 mg/kg/day, PO)

The selection of saroglitazar and fenofibrate doses was based on documented literature (12, 23, 24). The drugs were solubilized in a carboxy methyl cellulose (CMC) solution with a concentration of 0.5% w/v. The medications were orally delivered with the help of a conventional orogastric cannula daily for a duration of 21 days. Both food and water were freely available. Body weight measurements were taken weekly. After 21 days, foods were removed from the cages 12 hours before the animals were euthanized. Blood samples were obtained from tail veins, and serum was separated for 15 min with a 3000 rpm centrifugation. Following blood collection, the rats were euthanized, and the kidney was promptly removed, washed with PBS, and preserved in a 10% formalin solution for histological analysis. 

***Measurements of fasting blood glucose (FBG)***

Weekly assessments of fasting blood glucose levels were collected by extracting blood samples from the lateral tail vein and analyzing them with a one-touch glucometer (Dr. Morepen glucometer, Solan, Himanchal Pradesh, India) throughout the treatment period.

***Assessment of the serum biochemical markers***

The serum blood glucose levels, creatinine, albumin, and BUN were quantified using commercially accessible standard kits, following the manufacturer’s recommendations.

***Determination of the urine biochemical parameters***

The urine levels of kidney damage molecule-1 and type IV collagen were measured using commercially available ELISA kits, following the manufacturer’s directions.

***Histopathological examination***

The kidney tissue was extracted and deposited in a solution of 10% formaldehyde in PBS with a pH of 7.4. The tissue was then immersed in paraffin, sectioned, stained with hematoxylin/eosin (H&E), and analyzed under a light microscope.

***Statistical analysis***

All data were expressed as the mean ± SEM. Body weight was evaluated for significant differences using Multivariate analysis of variance (MANOVA) and, subsequently, Bonferroni’s *post hoc* test (25). The remaining parameters were subjected to a single-way ANOVA analysis and then a multiple comparison test by Tukey. *P*<0.05 was deemed to be statistically significant. The statistical assessment was performed utilizing the Graph pad Prism 3.06 software (Graph Pad Software, San Diego, CA, USA).

## Results

***In-silico interaction of saroglitazar with Kim-1 and Type IV collagen***

**Figure 1:**
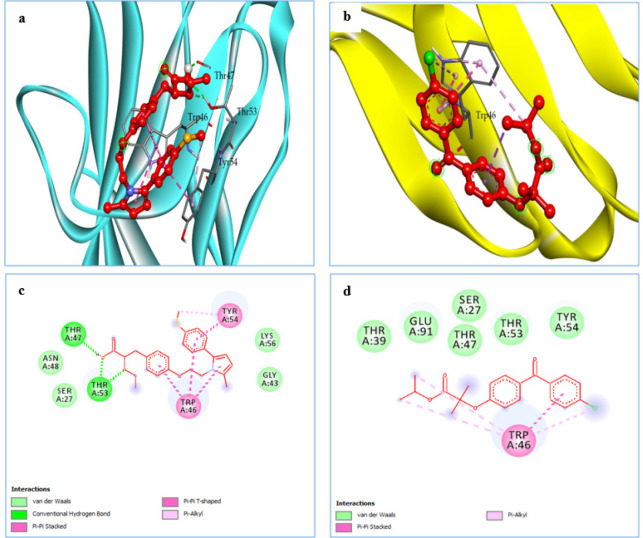
Binding mode and ligand interaction diagram of Saroglitazar (a), (c) and fenofibrate (b), (d), in the catalytic pocket of KIM-1 (PDB ID: 5DZO)

**Figure 2:**
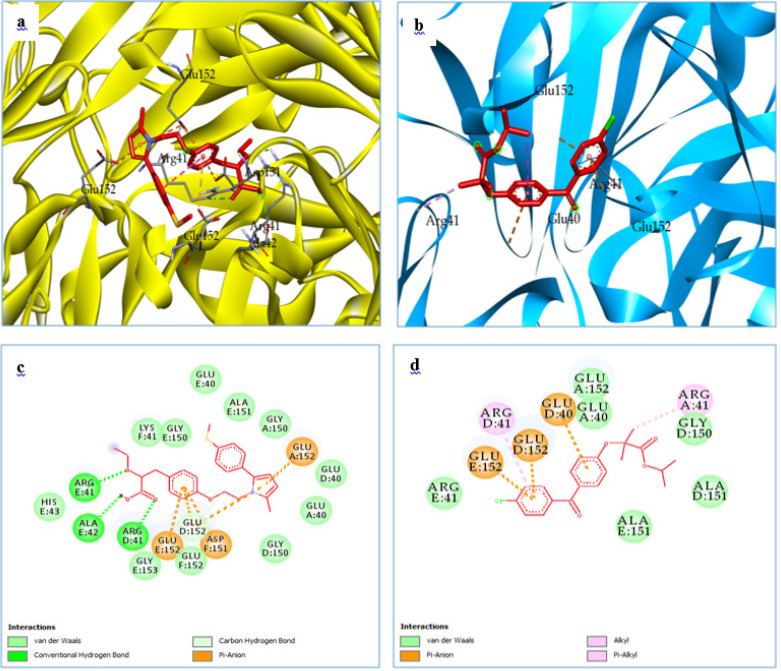
Binding mode and ligand interaction diagram of Saroglitazar (a), (c) and fenofibrate (b), (d), in the catalytic pocket of Type IV collagen (PDB ID: 1LI1)

The binding affinities of saroglitazar and fenofibrate to the active binding sites of KIM-1 (PDB ID: 5DZO) and Type IV collagen (PDB ID: 1LI1) are shown in Table 1. Molecular docking analysis revealed that the test drug, saroglitazar (Dock Score: -7.9 kcal/mol), displayed higher binding interactions with the ligand-binding domain (cavity) of Type IV collagen compared with the standard, fenofibrate (Dock Score: -7.2 kcal/mol). The size of the ligand binding cavity was 11.72 Å, 13 Å for 5DZO and 1LI1, respectively. Also, the binding affinity of saroglitazar (Dock Score: -5.3 kcal/mol) with KIM-1 was higher than fenofibrate (Dock Score: -5.1 kcal/mol). Figure 1 displays the two-dimensional interaction diagram of saroglitazar with the target Type IV collagen, whereas Figure 2 shows the three-dimensional interaction diagram of fenofibrate with the target KIM-1, respectively. Saroglitazar’s hydroxyl group established H-bonds with THR A: 47 and THR A: 53, while an O-atom of the ethoxy group produced an H-bond with THR A: 53 of KIM-1. Two of the aromatic rings displayed the pi- pi T-shaped interaction with TRP A: 46. One of the aromatic rings displayed the pi- pi T-shaped interaction with TYR A: 54. The hydrophobic pocket of KIM-1 was surrounded by the amino acid residues ASN A: 48, SER A: 27, LYS A: 56, and GLY A: 43. In the instance of Type IV collagen, the hydroxyl group of saroglitazar created a hydrogen link with ALA E: 42. Additionally, an O-atom of the ketone group made a hydrogen bond with ARG D: 41, and an O-atom of the ethoxy group produced a H-bond with ARG E: 41 of Type IV collagen. An aromatic ring exhibited a pi-pi T-shaped interaction with GLU E: 152, ASP F: 151, and GLU A: 152. The following residues generated the hydrophobic pocket of the functional region of Type IV collagen: HIS E: 43, GLY E: 153, GLU F: 152, GLY D: 150, GLU A: 40, GLU D: 40, GLY A: 150, ALA E: 151, GLU E: 40.

***Effect of saroglitazar on b.w. (gm/kg)***

In the present study, the HFD and low-dose STZ- administered rats (diabetic rats) displayed a significant increase (*P*<0.0001) in b.w., in comparison with normal control rats. Saroglitazar treatment (2 mg/kg, PO, 4 mg/kg, PO) respectively significantly reduced (*P*<0.0001) body weight in HFD and low-dose STZ-administered rats. Treatment with fenofibrate (100 mg/kg, PO) reduced the b.w. in HFD, and low-dose STZ was also administered to rats (Table 2).

***Effect of saroglitazar on serum blood glucose (mg/dl)***

**Figure 3:**
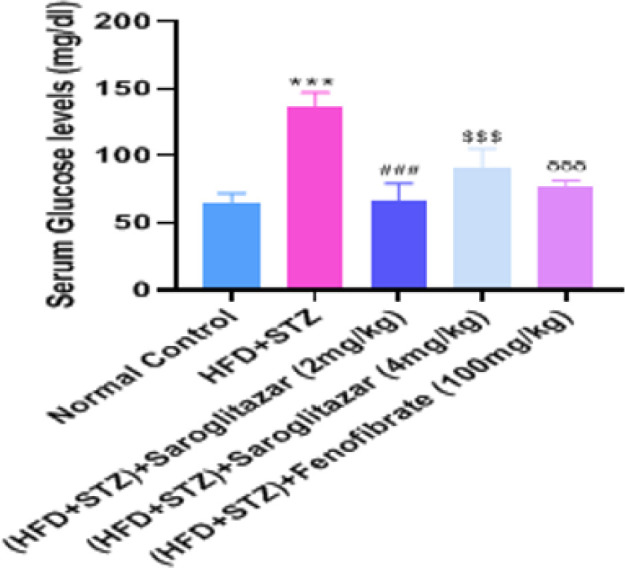
Effect of saroglitazar on serum glucose levels in HFD+STZ-induced type 2 diabetic nephropathy in Wistar rats

The HFD and low-dose STZ-administered rats showed a significant increase (*P*<0.0001) in the concentrations of serum blood glucose in comparison to normal control rats. Treatment with saroglitazar (2 mg/kg, PO, 4 mg/kg, PO) respectively caused a significant decrease (*P*<0.0001) in serum glucose level as compared to diabetic control rats. Fenofibrate treatment (100 mg/kg, PO) significantly reduced (*P*<0.0001) the serum glucose levels as compared to the diabetic control rats (Figure 3).

***Effect of saroglitazar on serum albumin (g/dl)***

The HFD and low-dose STZ-administered rats displayed significant decreases (*P*<0.0001) in serum albumin concentrations compared to normal control rats. Treatment with saroglitazar (2 mg/kg, PO 4 mg/kg, PO) respectively produced a significant increase (*P*<0.0001) in the serum albumin levels as compared to the diabetic control rats. Treatment with fenofibrate (100 mg/kg, PO) significantly elevated (*P*<0.0001) the serum albumin levels as compared to the diabetic control rats (Table 3).

***Effect of saroglitazar on serum creatinine (mg/dl)***

The HFD and low-dose STZ diabetic rats demonstrated a significant increase (*P*<0.0001) in serum creatinine levels compared to the normal control rats. Treatment with saroglitazar (2 mg/kg, PO and 4 mg/kg, PO) respectively produced a significant (*P*<0.0001) decrease as compared to diabetic control rats. Treatment with fenofibrate (100 mg/kg, PO) produced a significant reduction (*P*<0.0001) in the levels of serum creatinine as compared to the diabetic control rats (Table 3).

***Effect of saroglitazar on BUN level (mg/dl)***

The HFD and low-dose STZ diabetic rats displayed significant increases (*P*<0.0001) in the levels of serum BUN in comparison with normal control rats. Treatment with saroglitazar (2 mg/kg, PO and 4 mg/kg, PO) correspondingly caused a significant decrease (*P*<0.0001) in levels of serum BUN as compared to the diabetic control rats. Treatment with fenofibrate (100 mg/kg, PO) significantly decreased (*P*<0.0001) the levels of serum BUN as compared to the diabetic control rats (Table 3).

***Effect of saroglitazar on KIM-1 levels (pg/ml)***

**Figure 4:**
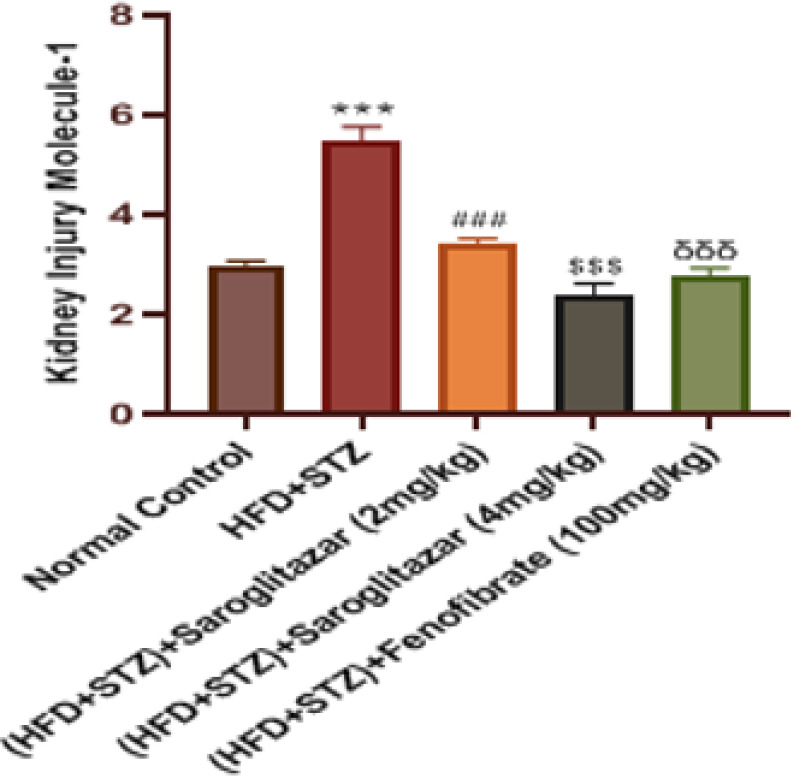
Effect of saroglitazar on urine KIM-1 in HFD+STZ-induced type 2 diabetic nephropathy in Wistar rats

The HFD and low-dose STZ diabetic-induced rats displayed a significant increase (*P*<0.0001) in the levels of KIM-1 in their urine as compared to the normal control rats. Treatment with saroglitazar (2 mg/kg, PO and 4 mg/kg, PO) produced a significant decrease (*P*<0.0001) in the levels of KIM-1 as compared to the diabetic control rats. Treatment with fenofibrate (100 mg/kg, PO) also significantly reduced (*P*<0.0001) the levels of KIM-1 as compared to the toxic diabetic rats (Figure 4).

***Effect of saroglitazar on type IV collagen (ng/ml)***

**Figure 5:**
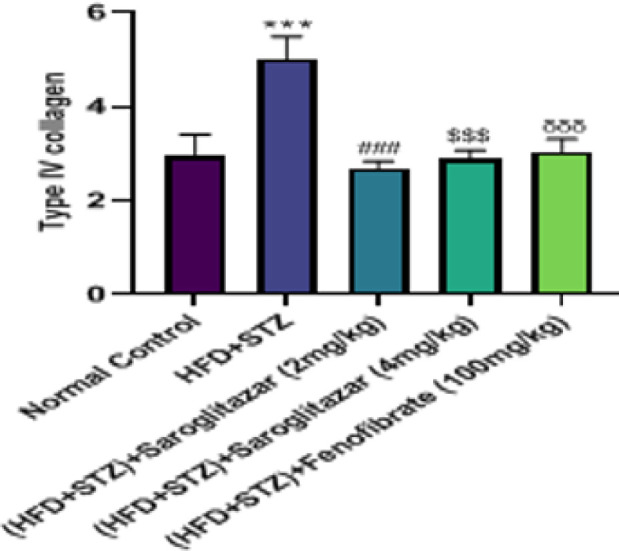
Effect of saroglitazar on urine Type IV collagen in HFD and low-dose STZ-induced type 2 diabetic nephropathy in Wistar rats

The HFD and low-dose STZ diabetic-induced rats displayed a significant increase (*P*<0.0001) in the concentrations of Type IV collagen in urine with a significant as compared with normal control rats. Treatment with saroglitazar (2 mg/kg, PO and 4 mg/kg, PO) produced a significant reduction (*P*<0.0001) in the levels of type IV collagen as compared to the toxic diabetic rats. Treatment with fenofibrate (100 mg/kg, PO) significantly decreased (*P*<0.0001) the levels of type IV collagen as compared to the toxic diabetic rats (Figure 5).

***Effect of saroglitazar on histopathological analysis of kidney***

**Figure 6:**
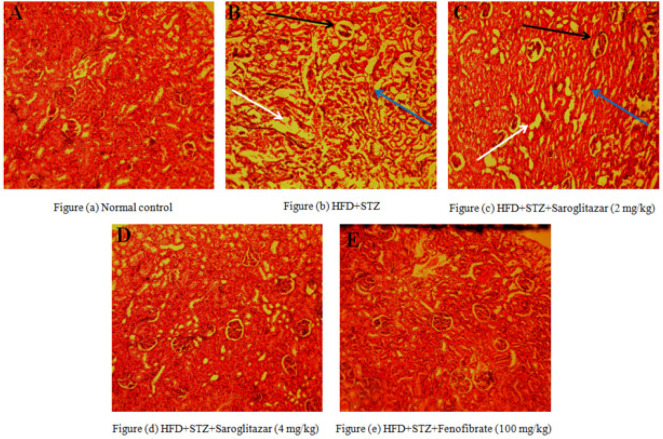
Photomicrographs consist of changes in the renal tissue of Wistar rats

Normal healthy control rats showed glomeruli and PCT in H&E-stained kidney specimens. HFD + STZ-treated rats showed tubular degeneration, necrosis, and dilatation of Bowman’s space. Treatment with saroglitazar (4 mg/kg, PO) and fenofibrate (100 mg/kg, PO) significantly reduced these histological changes, whereas saroglitazar (2 mg/kg, PO) was less significant (Figure 6).

## Discussion

The incidence and death rate linked to T2DM poses a significant global health concern. T2DM is closely linked to insulin resistance, obesity, and malfunction of the β-cells (26). DN is a term used to describe the occurrence of microvascular problems and end-stage renal disease in individuals with diabetes. It accounts for around 50% of newly diagnosed cases (27). Patients with DM who have albuminuria, a reduced estimated glomerular filtration rate, or both are clinically and histologically diagnosed as DN. DN is distinguished by several features, including enlargement of the glomeruli, expansion of the mesangial area in a diffuse or nodular pattern, thickening of the GBM, scarring of the glomeruli, loss of podocytes, damage to endothelial cells, atrophy of the tubules, fibrosis in the interstitial tissue, deposition of hyaline material in the arterioles, and infiltration of immune cells (28). 

Molecular docking is a computational method used to forecast the strength, binding affinity, and ideal positioning of a pharmacological molecule within the targeted catalytic binding region of the specific receptor (29). The binding pocket of the receptor is non-polar in nature and showed a better binding affinity with the non-polar ligands. Hence, the present study utilized molecular docking techniques to forecast the interaction between saroglitazar and fenofibrate with the ligand-binding site of KIM-1 and Type IV collagen. Docking analysis demonstrated that saroglitazar had a greater binding affinity with Kim-1 than fenofibrate, as shown by a Dock Score of -7.9 and -7.2, respectively. Moreover, saroglitazar exhibited notable binding interactions with the ligand-binding domain of type IV collagen, similar to the standard fenofibrate. Hence, this study demonstrates that saroglitazar can halt the advancement of DN by boosting renal function and improving histological renal alterations in diabetic rats. This effect is achieved through the involvement of KIM-1 and Type IV collagen proteins, which have not been previously shown. 

In this study, rats were fed an HFD for five weeks. On the 15^th^ day, they were administered a low dosage of STZ at a concentration of 35 mg/kg, IP. This rat model exhibited elevated blood sugar levels (hyperglycemia), high levels of lipids in the blood (hyperlipidemia), and reduced responsiveness to insulin (insulin resistance). These metabolic characteristics closely resemble those observed in humans with T2D (21). Further, Danda *et al.* (2005) (30) demonstrated that rats with T2D, generated by a diet high in fat and low-dose of STZ, experienced more severe kidney damage compared to rats with T1D, induced by a high dose of STZ injection. Therefore, this experimental paradigm is suitable for studying the impact of various treatment agents on DN. The Wistar rats exhibited insulin resistance syndrome, defined by a notable rise in body weight, slight elevation in blood glucose levels, increased levels of triglycerides and cholesterol, and compensatory hyperinsulinemia. This condition closely resembles the prediabetic, insulin-resistant state observed in people (31, 13). 

In 2005, Srinivasan *et al*. reported that the combination of HFD and low-dose STZ (35 mg/kg, IP) treated rat can be used as an alternative animal model. to stimulate T2D in humans because of IR and obesity. Using the methodology of Srinivasan *et al.*, 2005, a rat model T2D was successfully developed in the present study, which showed metabolic changes such as hyperglycemia, hyperlipidemia, impaired glucose tolerance, and decreased insulin sensitivity.

We investigated the effect of saroglitazar on HFD+STZ-induced diabetic rats at 2 and 4 mg/kg body weight for 21 days, producing a significant decline in body weight gain. It is fascinating and notable that the development of diabetes occurs only in insulin-resistant HFD and low-dose STZ (35 mg/kg, IP). Apart from glucose, HFD and insulin-resistant STZ animals also demonstrated abnormalities in renal function as evidenced by increased levels of serum blood glucose, albumin, creatinine, and BUN in the present study, as in the case of human T2D patients who may contribute to the end-stage renal disease i.e., DN.

Based on the existing literature review, we selected two markers of diabetes as possible biomarkers of DN: Kim-1 and type IV collagen. In this pre-clinical investigation, we examined whether the alterations of these two urine biomarkers were associated with DN and chronic renal scarring in a rat model.

Tubulointerstitial damage is a prevalent process of kidney disease progression in which the renal tubular cells have a vital function (32). Remarkably, kidney tubules are exceptionally able to undergo self-healing and regeneration following injury. KIM-1 is a protein found on the surface of cells that line the renal tubules. It is considered a biomarker since its levels increase when these cells are damaged (33, 34). Prior studies utilizing rat models have demonstrated that KIM-1 is a highly effective biomarker for kidney injury and surpasses blood creatinine as a superior predictor of kidney injury (33).

The current investigation found that rats given HFD+STZ showed a considerable rise in the concentration of KIM-1, while the saroglitazar treatment group of the Wistar rats showed a decreased level of KIM-1, which was not reported earlier.

Type IV collagen is the main constituent of the glomerular basement membrane (GBM) and mesangial matrix. Its presence in urine can serve as an early indication of renal impairment related to DN. Research conducted on animal models of experimental and genetic diabetes has shown that excessive production of type IV collagen causes the enlargement of the glomerular extracellular matrix, which results in a decrease in the filtration surface area (35, 36). 

One study revealed that individuals with diabetes in the normoalbuminuric group had significantly elevated levels of urine type IV collagen compared to the control group. Another investigation discovered that individuals with microalbuminuria had elevated urine type IV collagen levels compared to those with normoalbuminuria. Furthermore, these levels were found to be directly associated with the rate of urinary albumin excretion. This suggests that urinary type IV collagen could potentially serve as a valuable predictor for early DN in individuals with type diabetes (37, 38). Our research has revealed that urine type IV collagen concentrations were elevated in Wistar rats belonging to the HFD+STZ group. After the administration of saroglitazar, the elevated level of type IV collagen was significantly decreased.

Albumin is a singular protein species and the predominant plasma protein, constituting approximately 3/5 of the total quantity (39). Typically, the concentration of serum albumin in humans is approximately 45 g/L. Albumin is crucial in maintaining the oncotic pressure gradient between plasma and the interstitial space by controlling fluid exchange (40). Significantly, a decrease in blood albumin levels is linked to higher death rates (41).

Creatinine is readily available and commonly used as a biomarker to assess kidney function. It originates from creatine, a substance utilized in muscles as a rapid energy source. Creatine spontaneously and irreversibly transforms into creatinine, which is its anhydride form. Although creatinine is easily filtered and only marginally reabsorbed, the proximal tubule also releases approximately 20–30% of it (42). 

Creatinine is frequently employed as a metric to assess renal function. The typical creatinine clearance value is 110–150 ml/min and 100–130 ml/min for males and females, respectively (43). The National Kidney Disease Education Program advises the utilization of serum creatinine concentration to determine the glomerular filtration rate (44). Monitoring the development of renal disease is done with the creatinine clearance test. In our studies, we have found that serum creatinine levels were enhanced in HFD+STZ group Wistar rats. After treatment with saroglitazar, the increased level of serum creatinine was significantly reduced.

Urea, synthesized by the liver and distributed in both internal and extracellular fluid, is an important nitrogenous waste resulting from the breakdown of proteins and amino acids. The kidneys filter urea from the blood through the glomeruli and subsequently reabsorb it, along with water, to some extent (43). The primary factor used to determine clinical indicators for measuring renal function is the level of urea in serum. Measuring blood urea nitrogen-creatinine ratio is valuable in distinguishing between acute renal failure and pre-renal disorders (45). 

Higher blood urea nitrogen (BUN) levels are linked with renal illness or failure, urinary tract obstruction caused by renal stones, congestive heart failure (CHF), dehydration, fever, shock, and GI perforation. Elevated BUN levels may arise during the later stages of pregnancy or as a consequence of consuming significant quantities of protein-rich foods (46). In the present study, HFD+STZ-administered rats showed that BUN levels were significantly increased. The treatment group of the Wistar rats showed decreased levels of BUN which is not reported earlier.

The association between dyslipidemia and the occurrence of macrovascular problems in diabetes has been well-established for a considerable period of time (47). Moreover, research has demonstrated that DN is caused by increased buildup of lipids in the kidneys (48). Regulation of serum and urine KIM-1, type IV collagen leads to decreased risk of HFD and low-dose STZ-induced renal injury. Treatment with saroglitazar at specified dosages of 2 and 4 mg/kg orally resulted in significant improvements in serum glucose, albumin, creatinine, and BUN levels. Findings of the histological alterations in rat kidneys with T2D caused by an HFD and a low dose of STZ showed tubular degeneration, necrosis, and dilatation of Bowman’s space. 

Thus, these results suggested that saroglitazar would improve hyperglycemia and prevent diabetic complications. Precise evaluation of renal function is crucial due to diabetes being the leading factor behind chronic kidney disease (CKD). To illustrate a modification of renal function in diabetic rats, KIM-1 and type IV collagen levels were measured in urine. The present study showed that treatment with saroglitazar (2 and 4 mg/kg, PO) significantly prevented enhanced levels of KIM-1 and type IV collagen, essential biomarkers of renal disorder. The biochemical alterations were associated with a histopathological correlation assessment; High-fat diet (HFD) and low-dose STZ were found to cause significant kidney damage, including tubular degeneration, necrosis, and dilation of Bowman’s space.

 When administered orally at a dose of 4 mg/kg, saroglitazar was observed to be more efficacious in elevating albumin levels and decreasing serum glucose, creatinine, BUN, KIM-1, and type IV collagen levels in diabetic rats compared to saroglitazar (2 mg/kg, PO). 

**Table 1:** Dock score of saroglitazar and fenofibrate with the active binding site of kim-1 (pdb id: 5DZO) and type iv collagen (pdb id: 1LI1)

S. No.	Ligand	Dock score (kcal/mol)		Polarity
		KIM-1 (PDB ID: 5DZO)	Type IV collagen (PDB ID: 1LI1)	
1	Saroglitazar	-5.3	-7.9	1.90*10^-3^ mg/ml
2	Fenofibrate	-5.1	-7.2	2.14*10^-3^ mg/ml

kcal/mol: kilocalories per mole; KIM-1: kidney injury molecule; mg/ml: milligrams per milliliter

**Table 2:** Effect of saroglitazar on body weight in hfd and low dose stz- induced type 2 diabetic nephropathy in Wistar rats

Groups No.	Treatment	Baseline	Wk 1	Wk 2	Wk 3	Wk 4	Wk 5
I	NC	119.66±49.04	146.5±60.04	167.66±68.71	183.33±75.13	200.33±82.10	227.33±93.16
II	HFD+STZ	101.5±41.59^***^	126.83±51.98^***^	153.83± 63.04^***^	164.66±67.48^***^	178.66±73.22^***^	200±81.96^***^
III	HFD+STZ+Saroglitazar (2 mg/kg p.o.)	152.5±62.5^###^	175.83±72.06^###^	201.33±82.51^###^	208.16±85.31^###^	218.83±89.68^###^	231±94.67^###^
IV	HFD+STZ+Saroglitazar (4 mg/kg p.o.)	91.5±37.5^$$$^	115.16±47.19^$$$^	144.16± 59.08^$$$^	150.66±61.74^$$$^	171.16±70.15^$$$^	188.83±77.39^$$$^
V	HFD+STZ+Fenofibrate (100 mg/kg p.o.)	157±64.34^δδδ^	176.66±72.40^δδδ^	205.16± 84.08^δδδ^	206.33±84.56^δδδ^	215.66±88.38^ δδδ^	224.83±92.42^ δδδ^

All values were expressed as Mean ± SEM, (n=6); NC: normal control; HFD: high-fat diet; STZ: streptozotocin; mg/kg: milligrams per kilogram; PO: per ora;l Wk: week.

***P<0.0001, Gp I vs Gp II, ###*P*<0.0001, Gp II vs Gp III, *P*<0.0001, Gp II vs Gp IV, *P*<0.0001, Gp II vs Gp V

**Table 3:** Effect of saroglitazar on serum biochemical markers in HFD and low-dose STZ-induced type 2 diabetic nephropathy nephrotoxicity in Wistar rats

Group No.	Treatment	Albumin (g/dl)	Creatinine (mg/dl)	BUN level (mg/ml)
I	NC	3.45 ± 0.12	0.30 ± 0.03	26.07 ± 0.32
II	HFD + STZ	2.45 ± 0.12^***^	0.60 ± 0.04^***^	38.25±2.43^***^
III	HFD + STZ + Saroglitazar (2 mg/kg P.O.)	3.29 ± 0.03^##^	0.23 ± 0.02^###^	11.68±1.71^###^
IV	HFD + STZ + Saroglitazar (4 mg/kg P.O.)	3.53 ± 0.06^$$$^	0.30 ± 0.02^$$$^	20.11±1.72^$$$^
V	HFD + STZ + Fenofibrate (100 mg/kg P.O.)	3.62±0.11 ^δδδ^	0.27 ± 0.02 ^δδδ^	22.23±2.33 ^δδδ^

All values were expressed as Mean ± SEM, (n=6); NC: normal control; HFD: high-fat diet; STZ: streptozotocin; mg/kg: milligrams per kilogram; PO: per oral; g/dl: grams per deciliter; mg/dl: milligrams per deciliter; mg/ml: milligrams per milliliter.

***P<0.0001, Gp I vs Gp II, #*P*<0.01, Gp II vs Gp III, ###*P*<0.0001, Gp II vs Gp III, *P*<0.0001, Gp II vs Gp IV, *P*<0.0001, Gp II vs Gp V

All values were expressed as Mean ± SEM, (n=6); HFD: high fat diet; STZ: streptozotocin; mg/kg: milligrams per kilogram; mg/dl: milligrams per deciliter; and the data were analyzed by one-way ANOVA followed by Tukey’s-Kramer Multiple Comparisons Test. HFD+STZ vs normal control ***P<0.0001, HFD+STZ vs HFD+STZ+Saroglitazar (2 mg/kg PO) ###*P*<0.0001, HFD+STZ vs HFD+STZ+Saroglitazar (4 mg/kg PO) *P*< 0.0001, HFD+STZ vs HFD+STZ+Fenofibrate (100 mg/kg PO) *P*<0.0001.

All values were expressed as Mean ± SEM, (n=6); HFD: high fat diet; STZ: streptozotocin; mg/kg: milligrams per kilogram and the data were analyzed by one-way ANOVA followed by Tukey’s-Kramer Multiple Comparisions Test. HFD+STZ vs normal control ***P<0.0001, HFD+STZ vs HFD+STZ+Saroglitazar (2 mg/kg PO) ###*P*<0.0001, HFD+STZ vs HFD+STZ+Saroglitazar (4 mg/kg PO) *P*<0.0001, HFD+STZ vs HFD+STZ+Fenofibrate (100 mg/kg PO) *P*<0.0001.

All values were expressed as Mean ± SEM, (n=6); HFD: high fat diet; STZ: streptozotocin; mg/kg : Milligrams per kilogram and the data were analyzed by one-way ANOVA followed by Tukey’s-Kramer Multiple Comparisions Test. HFD+STZ vs normal control ***P<0.0001, HFD+STZ vs HFD+STZ+Saroglitazar (2 mg/kg PO) ###*P*<0.0001, HFD+STZ vs HFD+STZ+Saroglitazar (4 mg/kg PO) *P*<0.0001, HFD+STZ vs HFD+STZ+Fenofibrate (100 mg/kg PO) *P*<0.0001

A) Normal control rats showed glomeruli and PCT in normal renal. B) The HFD + STZ-treated group showed tubular degeneration, necrosis, and dilatation of Bowman’s space. C) HFD + STZ+ saroglitazar (2 mg/kg PO) group showed less significant changes as compared to saroglitazar (4 mg/kg PO). D) HFD + STZ + saroglitazar (4 mg/kg PO) treatment with saroglitazar (4 mg/kg) showed tubules and Bowman’s space without degeneration, necrosis, and dilatation. E) HFD + STZ+ fenofibrate (100 mg/kg PO) - showed a significant reduction in these histological changes. Blue arrows: tubular degeneration, White arrows: necrosis, Black arrows: dilatation of Bowman’s space.

HFD: high fat diet; STZ: streptozotocin

## Conclusion

The current research is the first to report on the impact of saroglitazar therapy on HFD+STZ-induced DN in Wistar rats. Saroglitazar treatment significantly reduced the b.w. gain, serum blood glucose, albumin, creatinine, and BUN in treated rats. Saroglitazar also lowered the KIM-1 and type IV collagen, improving renal activity and ameliorating renal histological changes in Wistar rats. These findings demonstrate that saroglitazar can serve as a highly efficient remedy for diabetes and its related kidney disease, nephropathy.
